# Bisphenol A and Bisphenol S Induce Endocrine and Chromosomal Alterations in Brown Trout

**DOI:** 10.3389/fendo.2021.645519

**Published:** 2021-03-11

**Authors:** Giada Frenzilli, Joan Martorell-Ribera, Margherita Bernardeschi, Vittoria Scarcelli, Elisabeth Jönsson, Nadia Diano, Martina Moggio, Patrizia Guidi, Joachim Sturve, Noomi Asker

**Affiliations:** ^1^ Department of Clinical and Experimental Medicine, Unit of Applied Biology and Genetics, University of Pisa, Pisa, Italy; ^2^ Institute for Genome Biology, Fish Genetics Unit, Leibniz Institute for Farm Animal Biology (FBN), Dummerstorf, Germany; ^3^ Department of Biological and Environmental Sciences, University of Gothenburg, Gothenburg, Sweden; ^4^ Department of Experimental Medicine, University of Campania “L. Vanvitelli”, Naples, Italy

**Keywords:** bisphenol, endocrine disruptor, genotoxicity, cell proliferation, *Salmo trutta*

## Abstract

Bisphenol A is a widely used compound found in large amount of consumer products. As concerns have been raised about its toxicological and public health effect, the use of alternatives to bisphenol A are now increasing. Bisphenol S is one of the analogues being used as a replacement for bisphenol A despite the fact that little is known about the effects of bisphenol S on living organisms. In this study, we investigated the potential endocrine and genotoxic effects of bisphenol A and bisphenol S in juvenile brown trout (*Salmo trutta*). The fish were exposed to the compounds for either 2 weeks or 8 weeks *via* sustained-release cholesterol implants containing doses of 2 mg/kg fish or 20 mg/kg fish of the substances. The effects on the thyroid hormone levels and the estrogenic disrupting marker vitellogenin were evaluated, along with the genotoxic markers micronucleated cells and erythrocyte nuclear abnormalities. An increase in plasma vitellogenin was observed in fish exposed to the high dose of bisphenol A for 2 weeks. At this experimental time the level of the thyroid hormone triiodothyronine (T3) in plasma was elevated after bisphenol S exposure at the high concentration, and paralleled by an increase of micronucleated cells. Moreover, bisphenol A induced an increase of micronuclei frequency in fish erythrocytes after the exposure at the lowest dose tested. Taken together the results indicate that both bisphenol A and its alternative bisphenol S cause endocrine disrupting and genotoxic effects in brown trout, although suggesting two different mechanisms of damage underlying bisphenol A and bisphenol S activity.

## Introduction

Chemicals released into the environment can mimic natural hormones affecting endocrine functions of animals and humans (1), thus acting as endocrine disrupting chemicals (EDCs). Such EDCs may represent a major toxicological and public health issue (2). The xenoestrogen bisphenol A (BPA) as many other plasticizers, have documented endocrine disruptive effects and has received much attention due to its high production volume and widespread human exposure (3). Indeed, BPA has also been detected in a variety of environmental samples, including water, dust, sewage, and indoor and outdoor air samples (4). The release of BPA from polycarbonates is accelerated by UV light, aging, heating, and contact with acidic or basic compounds (5, 6). Moreover, it can leach from plastic products, food and drink packaging thus contaminating canned food (5, 7, 8). The BPA exposure is estimated to be from 0.48 to 1.6 µg/kg body mass per day, for adults and children, respectively (9). The global distribution of BPA is now extent but it has been found that the bioaccumulation factor (BCF) is moderate (10) and it has been discussed whether or not BPA reach high enough concentrations in organisms to have biological effects. However, a large amount of studies, mainly laboratory studies, have demonstrated a long range of biological effects following BPA exposure. 

BPA has effects on both thyroid and estrogenic hormone systems in different experimental models (11, 12). Thyroid hormones, triiodothyronine (T3) and thyroxin (T4), have a key role in development, somatic growth, osmoregulation, and larval to juvenile transition or metamorphosis (13, 14). In mammals, the T3 and T4 secretion and its concentration in blood is regulated by the brain-pituitary-thyroid axis. In fish on the other hand, the brain-pituitary-thyroid axis mainly controls the release of T4 by the thyroid follicles and T4 homeostasis, while T3 activation and homeostasis is regulated by enzymatic de-iodination of T4 by deiodinases in the peripheral tissues, together with its release into the bloodstream (15–17). BPA interferes with the thyroid system at various levels, *e.g.* acting as antagonists for the thyroid receptors in the cell membrane, affecting the expression of thyroid hormone related genes, and/or affecting the development of the thyroid gland (11). A low exposure to BPA accelerated embryonic development and advanced the hatching in medaka fish (*Oryzias latipes*) through its effect on the thyroid receptor (18). In other fish species, disruption of the thyroid system by BPA has manifested itself in delayed hatching (19, 20), or impaired development and growth in fish; *e.g.* decreased tail length and body weight (21, 22). BPA may also act as a xenoestrogen in fish inducing the levels of vitellogenin (VTG) in both male and juvenile fish (23, 24). VTG is the main lipoprotein involved in the yolk synthesis in developing oocytes and necessary for embryogenesis and is usually expressed in sexually mature female fish (25). VTG is synthesized in the liver as a response to the activation of the estrogen receptor (ER) by estrogen or estrogen like substances (so-called xenoestrogens) (25). Increased VTG levels in male or juvenile fish is used as an indicator of estrogen like chemical exposure (25, 26). 

Mutagenic effects of BPA have also been reported, both genotoxic (27–29) and non-genotoxic (30, 31). DNA and chromosomal damage are the most important critical events following the exposure to carcinogenic and/or genotoxic agents. Various studies have strongly suggested that DNA damage induced by xenoestrogens and estrogen is dependent on estrogen receptors (ERs) (4, 32, 33). An *in vitro* study has indicated the effect of estradiol, an estrogen steroid hormone, on radiation-induced chromosome aberrations in human peripheral lymphocytes (34). Ionizing radiation (IR) and xenoestrogens, including BPA, are widely present in the environment and may act together, especially at low doses. In some studies, BPA was evaluated for its ability to induce micronuclei (MN), a well-known effect related to IR (35, 36), in human lymphoblastoid cell line MCL5 (37), human lymphoblastoid cell line AHH-1, and Chinese hamster V79 cells (38). 

Because of its negative effects on animal and human health, the use of BPA has been restricted in some countries (39–41), and instead new, alternative compounds, are being used to substitute BPA in the production of polymers (as well as a raw material in the preparation of a number of products) (42). Bisphenol S (BPS), a compound with more stability against high temperature and resistance to sunlight than BPA, is one of the substances being used a substitute to BPA in industrial applications (43, 44). BPS is commonly used in epoxy glues, canned foodstuffs, thermal receipt papers, paper currencies, luggage tags, food cartons, flyers, newspapers, etc. (44, 45). Due to the increasing use of BPS as a substitute for BPA, the environmental distribution of BPS in now increasing (46). Similar to BPA, BPS can act as an estrogen mimic or anti-androgen (47–50). Therefore, it is likely that BPS has the potential to interfere with, and disrupt the normal functions of endocrine system in organisms (51). Moreover, the genotoxic potential of BPS is also cause of concern since scientific literature reports conflicting results depending on the investigated experimental model (52, 53). As the usage of BPS as a substitute to BPA is increasing there is an urgent need to improve our knowledge about possible adverse effects of BPS on physiological functions in living organisms. 

The aim of the present study was to investigate adverse biological effects of BPS in comparison with BPA. This was done in order to evaluate BPS suitability as a substitute to BPA as a plasticizer. Juvenile brown trout (*Salmo trutta*) were exposed to both BPS and BPA for 2 and 8 weeks. The chemicals were administered through sustained-release cholesterol implant injected into the abdomen of the fish, in order to obtain a constant and low exposure, with the benefit of mimicking a chronic exposure with as little disturbance to the fish as possible. Endocrine effects were evaluated through levels of thyroid hormones, glucose and vitellogenin assessment while genotoxic effects were measured through micronucleated cells and nuclear abnormalities detection.

## Materials and Methods

### Chemicals

Implants: cholesterol, bisphenol A (CAS number 80-05-7) and bisphenol S (CAS number 80-09-1) were purchased from Merck KGaA (Darmstadt, Germany). VTG ELISA: secondary antibodies including horseradish peroxidase kit were obtained from Bio-Rad (Hercules, CA, USA). RIA: T3, and T4 label were purchased from Perkin-Elmer (Waltham, MA, USA) and antibodies for T3 and T4 were obtained from In Vitro technologies (Melbourne, Australia).Giemsa was purchased from Titolchimica spa (Rovigo, Italy).

### Fish Exposure

Juvenile individuals of *Salmo trutta* of both sexes at an approximate age of 12 months (average weight of 132 ± 25 grams) from Vänneåns fiskodling, Laholm, Sweden, were purchased and stored in two 600 L freshwater flow-through tanks for an acclimation period of 2 weeks in the animal facility of the Department of Biological and Environmental Sciences at the University of Gothenburg. Fish were kept at 10°C and photoperiod was 12/12 h of light/dark. After 2 weeks, animals were randomly distributed among ten 50 L aquaria (n = 10 per tank) with recirculation and aerated water. At the start of the experiment, each fish was carefully netted and anaesthetized (2-phenoxyethanol, 0.4 ml/L; ICN Biomedicals Inc., Costa Mesa, USA) before they were weighed and measured. The fish was then injected with a cholesterol implant of 20 mg weight containing either BPA or BPS (2 mg/kg fish or 20 mg/kg fish) or a sham implant with no chemical as control, into the abdominal cavity *via* a sharp incision made by a scalpel (54). The implant was administrated along with Passive Integrated Transporters (PIT-tags) with a unique barcode for individual identification of the fish. There were two replicate groups for each compound and dose (high and low dose BPA, high and low dose BPS, control). The concentration of BPA and BPS were based on previous toxicity data (data not shown). Photoperiod and temperature were kept constant during the whole experiment. The fish were fed a ratio of 1% of the body weight with commercial dry food (Biomar, Aarhus, Denmark) three times a week during both acclimation and exposure period. The study was performed under the ethical permit (274–2011) issued by the regional committee for animal experiments following national and European ethical guidelines and legislations on animal experimentation under the EU directive 2010/63/EU.

### Sampling

Sampling was performed 2 weeks and 8 weeks after the exposure. The sampling time points were selected to study effects after a shorter and longer exposure time of BPA/PBS, keeping within the time frame earlier used for sustained-release of hormone from cholesterol implants in fish (55). From each aquaria and time point, five fishes were sampled, for a total of 10 specimens *per* experimental point, except for four aquaria were fish died before sampling (1 dead fish in BPA high before the 2 weeks sampling, 2 + 1 dead fish in BPS low before the 8 week sampling, and 4 fish in one of the control aquaria before the 8 week sampling). Fishes were sacrificed with a sharp blow to the head and blood was immediately extracted from the caudal vein with a heparinized syringe. ID tag number, weight (g), and fork length (cm) were noted for each fish. Using the freshly extracted blood, hemoglobin and glucose levels were measured using a cuvette system from Hemocue (Ängelholm, Sweden) with assayed hemoglobin (HemoTrol) from Eurotrol (Ede, Netherlands) and glucose (GlucoTrol-AQ) from Eurotrol as quality controls. Part of the blood was centrifuged in a hematocrit capillary centrifuge for 2 min using capillary tubes to obtain the hematocrit level, and part of full blood was smeared on glass slides for the Cytome-Micronucleus assay. The main part of the extracted blood was centrifuged at 6,000 rpm for 5 min and the plasma fraction and stored in −80˚C until analysis. For each fish, liver was excised and weighed. Specific growth rate for length (SGRL) and weight (SGRW) were calculated for the two exposure times (2 weeks and 8 weeks). The implanting day was regarded as starting point of the experiment (length = L1, and weight = W1) and the sampling days were regarded as end point (length = L2, and weight = W2). Specific growth rate was calculated using the formula ln(W2/W1) × 100/days of exposure for SGRW and ln(L2/L1) × 100/days of exposure for SGRL. The condition factor (CF) for each individual was calculated using CF = weight(W2) (g)/length(L2)^3^ (cm) × 100 and liver somatic index (LSI) was calculated using LSI = liver weight (g)/body weight(L2) (g) × 100.

### Extraction and Clean-Up of Liver Samples

Bisphenols extraction from liver and quality control samples was performed in two steps: the liquid-liquid extraction of BPA and BPS with MeOH (1:1, v/v), and the solid phase extraction by AFFINIMIP^®^ SPE (Petit Couronne, France) columns. Therefore, 0.15 ± 0.02 g of liver tissue were mixed with 1.5 ml MeOH, containing 100 μl intermediate IS solution at 1 μg/ml and homogenized (3 min at 15,000 rpm) by using a Diax 900 homogenizer (Heidolph Instruments Gmbh & Co. KG, Schwabach, Germany). The homogenate was stirred (30 min) at room temperature and then centrifuged (10 min at 3,500 rpm) at 24°C. The supernatant was recovered, filtered on Whatman n. 1 paper, and dried under a gentle stream of nitrogen gas at room temperature. The residue was dissolved in 3 ml of water/MeOH (50:50, v/v) and then applied to an AFFINIMIP Bisphenols SPE cartridge, previously conditioned with 5 ml of MeOH containing 2% acetic acid, 5 ml ACN, and 5 ml of water, following the manufacturer instructions. After the sample loading, the cartridges were washed with 2 ml of water and eluted with 1 ml MeOH. The eluent was collected and afterwards concentrated by Eppendorf Concentrator at 45°C, up to a final volume of approximately 0.5 ml. The sample was diluted to 1.0 ml final volume with water. A 20 μl aliquot of the sample extract was injected for LC/ESI-MS/MS analysis.

### Liquid Chromatography–Electrospray Ionization Tandem Mass Spectrometry Analysis

In this study, a Dionex UltiMate 3000 HPLC system (Thermo Fisher Scientific Inc, Monza BZ, Italy) was employed. The chromatographic separation was performed using a 50 × 4.6 mm Kinetex 2.6 μm F5 stainless steel HPLC column, equipped with a guard column with the same stationary phase (2 × 4.6 mm, particle size 2.6 μm) (Phenomenex, Bologna, Italy). A mobile phase of water (A) and MeOH (B), without additives, such as formic acid, acetic acid, ammonium acetate, or ammonia, was used. Chromatography was run at room temperature by linear gradient elution; the analysis begins with 30% (v/v) B for 1 min, then followed by a gradient from 30% (v/v) to 95% (v/v) B in 4 min holding at 95% (v/v) B for 3 min. Finally, the mobile phase B was decreased to 30% (v/v) in 1 min and equilibrated at 30% (v/v) for further 3 min. The flow rate was 0.3 ml/min. The injection volume was 20 µl.

HPLC system was coupled to a triple quadrupole instrument (API 2000; AB Sciex, Germany) equipped with a TurboIon electrospray source. Analytes were detected in negative ion mode at a vaporization temperature of 450°C and an ion electrospray voltage of −4.5 kV. Other parameters were set at −40V for Declustering Potential (DP), −10V for Entrance Potential (EP), −35eV for Collision Energy (CE). A 6 psi N2 was used as collision gas, setting the curtain gas at 40 psi. The analytes were quantified in multiple reaction monitoring (MRM) mode. The transitions monitored in MRM mode for BPA were 227.1 > 212.1 and 227.1 > 133.2 m/z for its quantifier and qualifier ions, respectively, and 243.1 > 215.0 and 243.1 > 132.1 m/z for d_6_-BPA (internal standard). The transitions monitored in MRM mode for BPS were 249.0 > 108.0 and 249.0 > 92.0 m/z for its quantifier and qualifier ions, respectively. BPA and BPS identification was based on the retention time of both quantifier and qualifier product ions. The data were analyzed by the Analyst™ software version 1.5.1 (ABI Sciex).

### T3 and T4 RIA

The levels of total thyroid hormones triiodothyronine (T3) and thyroxine (T4) in blood plasma were quantified using a radioimmunoassay (RIA) protocol as described previously (56, 57). A test of parallelism with serial dilutions of plasma, was carried out to validate the RIA method for brown trout, and showed that the standard and plasma slopes were parallel. Each plasma sample was diluted 1:10 in RIA buffer for the assay, and each sample was run in duplicate tubes. Antibody (Ab) working solutions were made in RIA buffer with 1:144 dilution for T3Ab and 1:400 dilution for T4Ab.

### Vitellogenin ELISA

Plasma vitellogenin (VTG) levels were quantified using a competitive enzyme-linked immunosorbent assay (ELISA) as previously described for rainbow trout (58). Plasma samples were diluted 1:40 and incubated with rabbit anti-arctic char antibodies (PO-1), diluted 1:3,000 and thereafter added to VTG-coated plates. The level of VTG in each plasma sample was detected using horseradish peroxidase labeled goat anti-rabbit antibodies (diluted 1:2,000) and quantified with a standard curve with purified VTG.

### Cytome Assay

Peripheral blood samples obtained from the caudal vein of the specimens were processed for Cytome-Micronucleus assay according to Frenzilli and co-workers (59). Blood samples smeared on glass slides were fixated in pure ethanol for 20 min, the slides were allowed to air-dry and then the smears were stained with 10% Giemsa solution for 25 min. For each specimen two slides were set up, all the slides were coded and scored blindly. The frequency of micronuclei was estimated by scoring of 4,000 random erythrocytes (2,000/slide) from each individual. The number of analyzed animals was 5 (n = 5) for each treatment at both experimental exposure time except for low dose of BPS at 2 and 8 weeks where analyzed specimens were three and four, respectively. In addition to micronucleus frequency, the frequencies of other erythrocyte nuclear abnormalities, such as nuclear buds, blebbed, notched, lobed, circular nuclei, and nucleoplasmic bridges, and binucleated cell (a cell is considered binucleated when it has an intact cytoplasm and normal nucleus morphology containing one more nucleus), were evaluated (×100 magnification) (60–62). The different frequencies of these anomalies are related to specific genotoxic events associated to the different mechanisms of action of the carcinogenic/mutagenic agents. MN was selected as a marker of chromosomal breakage and/or loss, nucleoplasmic bridges as markers of chromosomal rearrangements, nuclear evaginations, or buds as markers of gene amplification (63).

Non-refractive, circular, or ovoid chromatin bodies, smaller than the one-third of the main nucleus, displaying the same staining and focusing pattern as the main nucleus and clearly separated from it were scored as micronuclei (64). They arise from chromosomal fragments or whole chromosomes that are not incorporated into daughter nuclei at mitosis and are index of chromosomal breakage or mitotic spindle dysfunctions (65).

### Statistical Analysis

For the statistical analysis a one-way ANOVA test (analysis of variances) was performed with SPSS statistics, separately for the 2 and 8 week exposures. Homogeneity of variances was checked by a Levene’s test and the variables which not fulfill this assumption were transformed to ln(x) prior the ANOVA test. Variables were considered significant with a 95% confidence interval (p-value < 0.05). Tukey’s *post-hoc* test was implemented for a multiple group comparison. A student t-test was performed to evaluate if there were any differences between high and low dose for BPA and BPS separately for the 2 and 8 weeks exposure. While VTG is a biomarker of exposure to estrogenic compounds, whose levels are *per se* informative, that is the reason why the Authors decide to apply one-way ANOVA test, the genotoxicity endpoints (MN, BN) used are biomarkers of effect, which can be influenced by experimental variables. For this reason, the multifactor analysis of variance (MANOVA) or Multiple Regression Analysis (MRA) were used, in order to take into consideration all parameters (independent variables) able to influence the mean and standard deviation (SD) of genotoxicity endpoints, and make the statistical approach more rigorous. Micronuclei and binucleated cells were considered as dependent variables; dose, experimental time, treatment, slide, scorer, hematological parameters, bisphenol concentrations in the liver as independent variables. The Multiple Range Test (MRT) was performed in order to detect differences among experimental groups. For all data analysis, statistical significance was set at p-value < 0.05.

## Results

### Biometric Data

Body weight, fork length, and liver weight noted for each fish during the implanting day or the 2 and 8 weeks sampling days did not significantly differ between groups (p > 0.05) (results not shown). The calculated SGRW, SGRL, CF, and LSI were similar among the treatment groups after 2 weeks of exposure (p > 0.05; **Table 1**). After 8 weeks, LSI was significantly higher in fish exposed to low dose of BPS compare to control fish.

**Table 1:** Biometric and hematological data in *Salmo trutta* exposed to high and low concentrations of BPA and BPS.

Exposure time	Treatment	SGRW	SGRL	CF	LSI	HT	Hb	Glu
		%	%	%	%	%	g/L	nmol/L
**2 weeks**	C	0.17 ± 0.16	0.05 ± 0.04	1.06 ± 0.02	1.33 ± 0.09	31.1 ± 1.3^ab^	77.0 ± 4.6	6.0 ± 0.5
	*n*	10	10	10	10	8	10	10
	BPA L	0.16 ± 0.16	0.04 ± 0.04	1.06 ± 0.03	1.40 ± 0.08	29.6 ± 0.9^ab^	73.2 ± 3.0	5.8 ± 0.3
	*n*	10	10	10	10	10	10	10
	BPA H	0.27 ± 0.21	0.11 ± 0.06	1.11 ± 0.05	1.51 ± 0.07	31.3 ± 1.6^ab^	77.2 ± 5.6	5.6 ± 0.3
	*n*	9	9	9	9	8	9	8
	BPS L	0.53 ± 0.23	0.02 ± 0.03	1.13 ± 0.06	1.36 ± 0.10	27.4 ± 2.3^a^	71.4 ± 6.0	4.6 ± 0.2
	*n*	10	10	10	10	10	10	10
	BPS H	-0.16 ± 0.22	0.03 ± 0.02	0.95 ± 0.11	1.23 ± 0.09	34.4 ± 1.4^b^	83.8 ± 4.9	5.7 ± 0.7
	*n*	9	10	10	8	10	10	10
**8 weeks**	C	0.15 ± 0.22	0.09 ± 0.06	0.98 ± 0.02	1.17 ± 0.09^a^	25.3 ± 1.2	57.5 ± 2.7	5.7 ± 0.5
	*n*	6	6	6	6	6	6	6
	BPA L	0.72 ± 0.20	0.06 ± 0.04	0.96 ± 0.03	1.30 ± 0.05^ab^	26.7 ± 1.5	59.2 ± 2.8	7.7 ± 1.1
	*n*	10	10	10	10	10	10	10
	BPA H	-0.03 ± 0.25	0.06 ± 0.06	0.96 ± 0.03	1.40 ± 0.12^ab^	24.4 ± 2.0	61.3 ± 3.7	7.5 ± 0.9
	*n*	9	9	9	9	9	10	8
	BPS L	0.11 ± 0.18	0.06 ± 0.05	0.97 ± 0.02	1.76 ± 0.11^b^	23.3 ± 2.5	56.6 ± 4.4	10.2 ± 1.5
	*n*	7	7	7	6	6	7	6
	BPS H	0.16 ± 0.22	0.09 ± 0.05	0.98 ± 0.02	1.30 ± 0.11^a^	27.4 ± 1.7	66.9 ± 5.3	8.3 ± 1.0
	*n*	10	10	10	10	9	10	10

Treatments were performed at different concentrations (L = 2 mg/kg; H = 20 mg/kg) of BPA and BPS, at two different times of exposure (2 weeks and 8 weeks). Values indicated for specific growth rate for weight (SGRW), specific growth rate from length (SGRL), condition factor (CF), liver somatic index (LSI), hematocrit (HT), hemoglobin (Hb), and glucose (Glu) at the sampling day. C, control group; and n, number of individuals. Assessments were performed on 46–49 individuals for the 2 weeks’ and 40–43 individuals for the 8 weeks’ exposure. Data shown as mean ± standard error. Letters indicate statistically significant differences between groups at 2 or 8 weeks separately (p < 0.05).

### BPA and BPS Bioaccumulation

The concentrations of BPA and BPS detected in fish liver tissues from both 2 and 8 weeks sampling were a magnitude of more than 2,500 times higher in BPA/BPS-exposed fish compared with controls, which had very low levels, confirming that the sustained-release implants were active during the whole experimental period (**Table 2**). A slightly but not significant higher concentration of the chemicals was noted in fish exposed to the higher dose of BPA and BPS for both 2 and 8 weeks of exposure (**Table 2**). In addition, a positive correlation was found between tissue concentration of bisphenols and treatment dose used, including control values both for BPA (p < 0.001) and for BPS (p < 0.001).

**Table 2:** BPA and BPS liver concentrations at different exposure times and exposure doses.

Exposure time	Treatment	Dose	Conc. in liver
		mg/kg	ng/g
**2 weeks**	C	0	0.02 ± 0.01^a^
	*n*		*10*
	BPA L	2	27.2 ± 17.7^b^
	*n*		*10*
	BPA H	20	42.2 ± 23.1^c^
	*N*		*9*
	BPS L	2	25.3 ± 11.1^b^
	*n*		*10*
	BPS H	20	28.5 ± 21.8^b^
	*n*		*10*
**8 weeks**	C	0	0.02 ± 0.17^a^
	*n*		*6*
	BPA L	2	37.6 ± 11.7^b^
	*n*		*10*
	BPA H	20	45.8 ± 12.7^c^
	*n*		*10*
	BPS L	2	33.4 ± 10.0^b^
	*n*		*7*
	BPS H	20	42.9 ± 15.3^c^
	*n*		*10*

Treatments were performed at different concentrations (L = 2 mg/kg; H = 20 mg/kg) of BPA and BPS, at two different times of exposure (2 weeks and 8 weeks). Concentration in liver (conc. in liver) at the time point of sampling is indicated in ng/g (wet weight). C, control group. Letters indicate statistically significant difference with respect to control within each group (MRT p < 0.05). n, number of individuals. Assessments were performed on 49 individuals for the 2 weeks’ and 43 individuals for the 8 weeks’ exposure. Data are shown as mean ± standard deviation.

### Hematological Data

Hematocrit and hemoglobin levels showed no statistical significant difference within BPA and BPS treatment groups in comparison with controls (p > 0.05; **Table 1**) except for a significantly higher hematocrit level in fish exposed to the high dose of BPS compared to fish exposed to the low dose of BPS after 2 weeks of exposure.

#### Glucose Levels in Blood Plasma

Glucose levels showed no significant difference in the BPA and BPS exposed fish after 2 weeks or 8 weeks compared to control. However, after 8 weeks, a higher glucose level was noted in the BPS high dose group (p = 0.084) groups as well as a slightly higher in BPA exposed fish compared to controls (**Table 1**). A statistically significant positive correlation was also found between BPA (p < 0.01) and BPS (p < 0.001) liver concentrations and glucose levels.

#### T3 and T4 Levels in Blood Plasma

**Figure 1:**
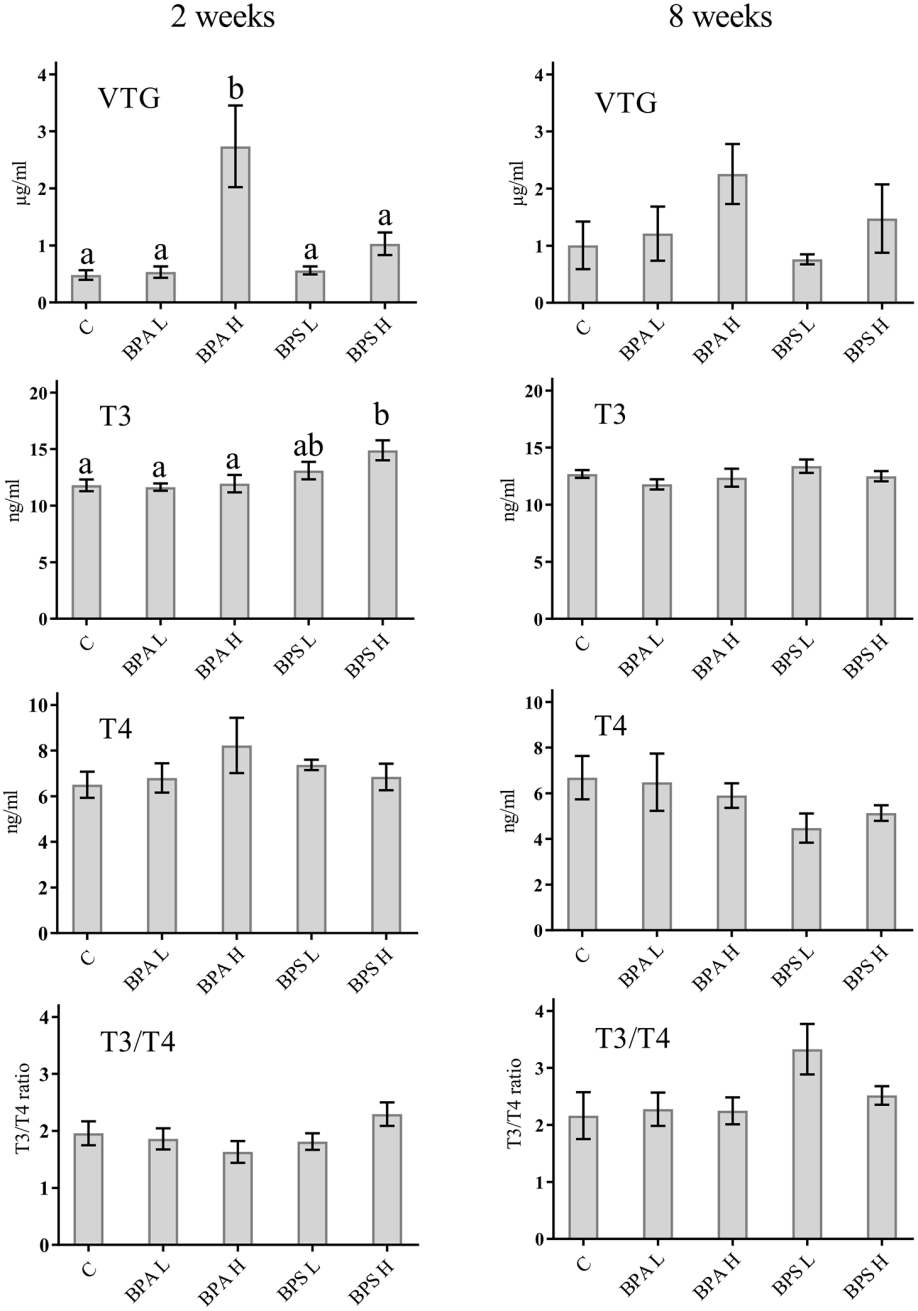
Levels of VTG, T3, and T4 as well as T3/T4 ratio in plasma from *Salmo trutta* shown as mean ± standard error. Treatments were performed at different concentrations (L = 2 mg/kg; H = 20 mg/kg) of BPA and BPS at two different times of exposure (2 weeks or 8 weeks). C, control group. Letters indicate statistically significant differences between groups at 2 or 8 weeks separately (p < 0.05). Assessments were performed on 9–10 individuals for the 2 weeks’ and 6–10 individuals for the 8 weeks’ exposure.

After 2 weeks, T3 levels were significantly higher in fish treated with the high dose of BPS compared with controls and BPA treated fish (**Figure 1**). There were no significant differences in plasma T3 levels among treatment groups after 8 weeks (**Figure 1**). Neither plasma T4 levels nor T3/T4 ratio differed significantly between treatment groups after 2 or 8 weeks of exposure.

#### Vitellogenin Levels in Blood Plasma

Fish exposed to a high dose of BPA showed a significant higher amount of VTG in plasma compared with control fish (p < 0.05) after 2 weeks of exposure (**Figure 1**). After 8 weeks of exposure to the higher concentrations of BPA, there was still a high amount of VTG in plasma, however not significant (**Figure 1**). VTG measurements after BPA exposure to low concentration for 2 or 8 weeks showed no difference compared with the control (**Figure 1**). Exposure to BPS at low concentrations did not indicate any significant difference compare to control fish for 2 or 8 weeks (**Figure 1**).

### Cytome Assay

**Figure 2:**
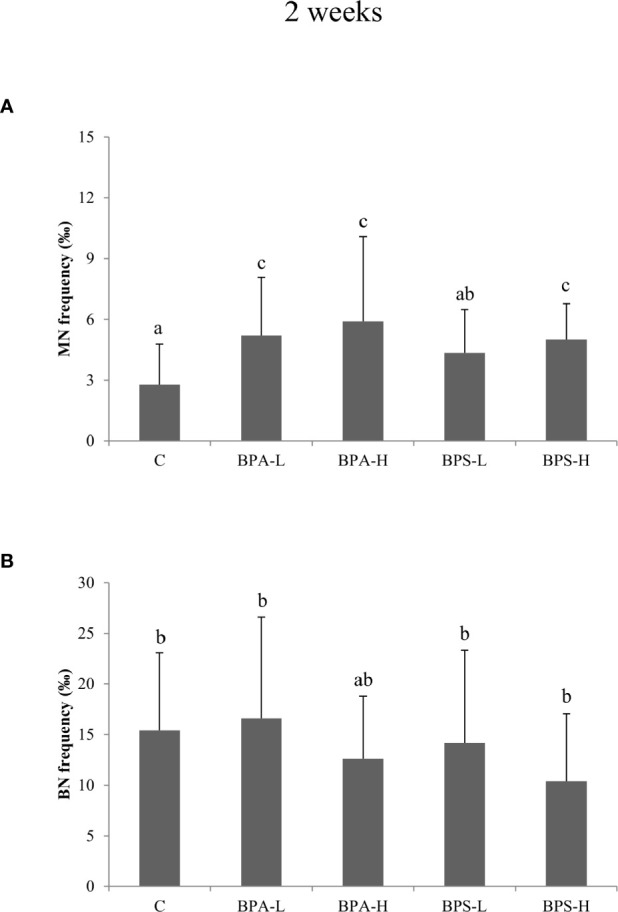
Frequency of micronucleated **(A)** and binucleated **(B)** cells in *Salmo trutta* erythrocytes shown as mean ± standard deviation. Treatments were performed at different concentrations (L = 2 mg/kg; H = 20 mg/kg) of BPA and BPS for 2 weeks of exposure. n = 5 for each experimental group except for BPS-L treatment group where n = 3. Assessments were performed on 23 individuals. C, control group. Letters indicate statistically significant differences between groups (ANOVA, Multiple Range Test, p < 0.05).

**Figure 3:**
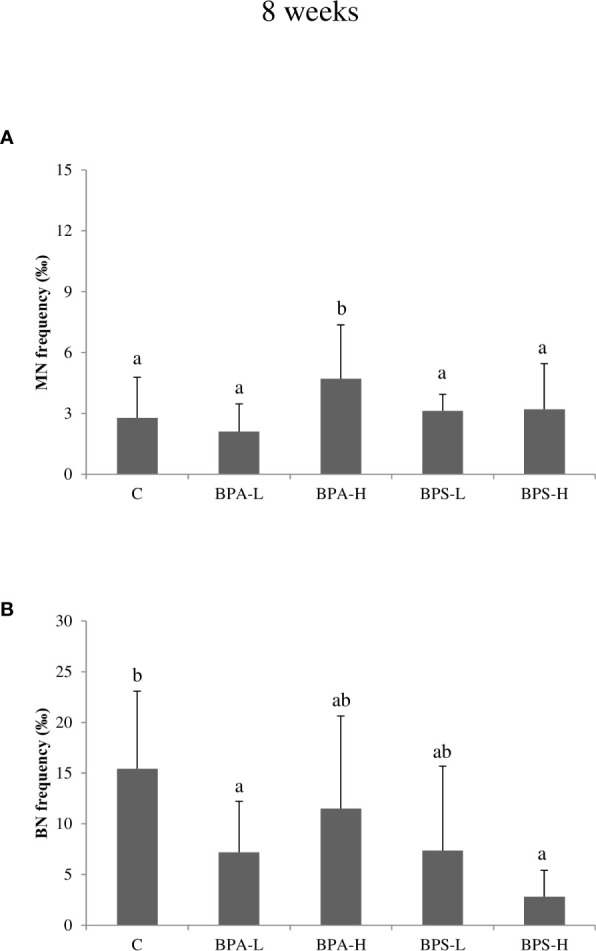
Frequency of micronucleated **(A)** and binucleated **(B)** cells in *Salmo trutta* erythrocytes shown as mean ± standard deviation. Treatments were performed at different concentrations (L = 2 mg/kg; H = 20 mg/kg) of BPA and BPS for 8 weeks of exposure. n = 5 for each experimental group except for BPS-L treatment group where n = 4. Assessments were performed on 24 individuals. C, control group. Letters indicate statistically significant differences between groups (ANOVA, Multiple Range Test, p < 0.05).

After 2 weeks BPA exposure, Cytome assay revealed a statistically significant increase (p < 0.05) in micronucleated (MN) cells after the exposure to both low and high dose (**Figure 2A**), and such an increase was statistically associated to BPA tissue concentration (p < 0.01). Moreover, a statistically significant (p = 0.02) effect of dose was detected, indicating higher frequencies of micronuclei in organisms exposed to the higher dose. No effects on cell cycle were observed (**Figure 2B**). After 8 week exposure, no chromosomal damage increase was assessed with respect to controls (**Figure 3A**). On the contrary, no significant differences were observed after 8 week exposure with respect to controls (**Figure 3A**). Looking at cycling cells, a decrease of binucleated cells (BN) frequency was shown after an 8 week treatment with the lowest BPA dose (**Figure 3B**).

The high BPS dose induced an increase in MN frequency after 2 week treatment (**Figure 2A**), while the other treatments showed DNA damage levels comparable to the control ones. A reduction in BN cells was found only at the highest dose after 8 week treatment (**Figure 3B**).

MANOVA showed a positive correlation between BPA and BPS liver concentrations and the frequency of micronucleated cells (p < 0.001) and to the incidence of total nuclear abnormalities (p < 0.01). Looking at metabolic parameters a weak but statistically significant positive association (p < 0.05) between BPA-induced micronucleated cells and blood glucose concentration was found. In BPS treated animals, a general negative correlation (p < 0.01) was found between MN frequencies and the incidence of nuclear structural abnormalities. Moreover, the variable BPS liver concentration explained more than 60% of the variance among nuclear abnormalities observed and a positive correlation (p = 0.001), was found with BPS liver concentration and bud frequency after 2 week exposure.

## Discussion

There is a big societal concern that bisphenol A (BPA) exerts harmful effects in a range of organisms, including endocrine disruptive and genotoxic effects. Moreover, there is almost a lack of information concerning the potential adverse effects of substitutes to BPA, such as bisphenol S (BPS), especially in the aquatic environment. Therefore, we compared biological effects of BPA and BPS in brown trout exposed to these compounds for 2 and 8 weeks using slow-release implants to attain a chronic exposure to these chemicals. The cholesterol pellet technique has been demonstrated to be an effective treatment method for chronic release of lipid- and water-soluble substances in fish in laboratory as well as field experiments at different temperatures (54, 66). The implant also has the advantage that the stress induced to the fish, and/or other negative side effects, by repeated injections to mimic chronic exposure is avoided (67). The measured levels of BPA and BPS in liver at the two sampling points confirm the usefulness of the exposure protocol during the whole experimental period, in line with previous results using this technique (66). However, results also show that the BPA or BPS levels in livers were similar regardless of exposure time or exposure level, even though the high dose exposed fish showed slightly elevated levels of the administered compounds. Although earlier studies reported a positive association between BPA tissue concentrations and used BPA dose in other vertebrate models (68), in the present study relatively low level of the chemicals found in the liver compared to the concentration in the implant. This is likely due to a continuous but slow release of chemicals from the implant in combination with a low tendency for bioaccumulation of bisphenols (9, 10) which is in accordance with the low log Kow estimated for BPA (3.64) and BPS (1.65) (69). The levels of BPA/BPS found in the brown trout liver after 2 and 8 weeks were similar to the concentration of BPA found in liver of field sampled fish from earlier studies (10, 70, 71). 

In general, studies addressing the endocrine effects of BPA and the more recently introduced BPS have mostly focused on potential androgenic/antiandrogenic and estrogenic actions (51). Nevertheless, there is an increasing number of studies showing that both BPA and BPS interfere with the thyroid system function through various mechanisms of action at different levels, both *in vivo* and *in vitro* (11, 72, 73). In the current study, however, BPA exposure had no effect on plasma T3 or T4 levels, or T3/T4 ratio, which may imply that BPA does not influence thyroid function in brown trout. This result is different from previous studies on zebrafish reporting variable responses in thyroid hormone levels as well as on gene expression of genes linked to thyroid function after BPA exposure (72). Although our data do not support that BPA interferes with T3 and T4 in brown trout, additional studies targeting different parts of the thyroid system should be done before definitive conclusions that BPA does not affect thyroid endocrinology are drawn. The high dose BPS, but none of the other BPA/BPS treatments, resulted in elevated T3 plasma levels after 2 but not after 8 weeks, indicating a time- and dose-dependent effect of this substance. This is similar to the effect observed in a recent study on adult zebrafish (74), but different from a study on zebrafish larvae, where both T3 and T4 levels were reduced after BPS exposure (75). In their study, Zhang et al. (75) also showed that BPS affected several genes in the HPT-axis, which was later also supported by a study on tadpoles *Pelophylax nigromaculatus* (76), emphasizing the many possible targets of actions endocrine disruptive substances may have. Indeed, varied results in terms of BPA as well as BPS effects on thyroid function may be due to life stage, treatment times, and route of exposure. Our data imply that BPS is more efficient in affecting thyroid hormone action than BPA, potentially acting at tissue deiodinase activity since only T3 levels, and not T4, changed. Hence, our study adds to the picture that BPS is a thyroid hormone disruptor, with potential effects on metabolism and development (51). This result is further supported by a recent study showing that BPS has transgenerational effects and interferes with development of zebrafish, along with effects on thyroid hormones (74).

VTG plasma levels have been widely used as an endpoint biomarker for exposure to estrogenic substances in fish (25). In the present study, a significant increase in VTG was observed after 2 weeks of exposure to BPA when the fish were treated with the high dose compared to low dose and control. These findings are in agreement with an earlier BPA exposure study, where female zebrafish showed a significant dose-dependent increase in whole body VTG after short-term water exposure (24). A similar result was observed in adult male and females of common carp (*Cyprinus carpio)* exposed to increasing water concentrations of BPA, where a significantly higher concentration of plasma VTG was detected at 1,000 µg/L (23). In contrast, adult female rare minnow (*Gobiocypris rarus)* exposed to low BPA concentrations (5, 15, and 50 µg/L) for 14 and 35 days, indicated a time-dependent rather than a dose-dependent increase of the potential endocrine effects of BPA when measuring hepatic VTG transcription (77). In the present study, the levels of VTG were only significantly higher after 2 weeks of exposure and not after 8 weeks. This may possibly indicate a different time- and dose-dependent effect of BPA in brown trout and/or different effect due to differences in routes of exposure. A number of studies showing that that BPA may act as an estrogen receptor (ER) agonist enhancing vitellogenesis and increasing plasma VTG support the estrogenic effect of BPA on juvenile fish (9, 78–80) and the results presented herein on juvenile brown trout, further strengthen the view that BPA has an endocrine disrupting and xenoestrogenic capacity in fish. Concerning potential estrogen-like action of BPS, Naderi et al. (81) exposed zebrafish embryos to increasing concentrations of BPS for 75 days *via* water exposure and detected a significant increase of plasma VTG in both male and female (81). This is in contrast to our study where no significant increase in VTG was noted in BPS exposed fish at any concentration or exposure time. In a recent study, BPA alternatives were investigated in a zebrafish embryo model to assess lethality, developmental effects, and estrogenic activity (82). The study showed that exposure to the bisphenols BPFA, BPA and BPF significantly elevated the transcription of genes coding for VTG and three estrogen receptors in the zebrafish embryos after 4 days of exposure, while BPS exposure exhibited no effect on the transcription of these genes. The authors concluded that BPS had the lowest acute toxic effect on zebrafish embryos and that BPAF had the highest toxic effects and estrogenic activity, followed by BPA and BPF (82). Hence, the present study seems to confirm such data depicting BPS as a thyroid hormone disruptor rather than a xenoestrogen in the experimental conditions used.

Measurement of glucose levels in plasma is a good indicator for glucose homeostasis, and skewed glucose levels may indicate disturbed insulin regulation (83). In the present study, fish from the both BPA and BPS treated groups had elevated glucose levels compared with control fish. Also looking at single specimens some of the fish demonstrated glucose levels outside the normal range, and glucose is a physiological parameter that should be regulated tightly. These results may indicate that BPS disturb the regulation of glucose homeostasis and/or metabolic effects. This is in line with the study by Zhao et al. (84) showing that BPS exposure increased blood glucose levels as well as lowered insulin levels in zebrafish. It has also previously been shown that carbohydrate pathways are affected in BPA exposed hepatocytes possibly leading to perturbations in glucose homeostasis in these cells (85). Hence, both BPS and BPA seem to affect glucose levels in fish through various mechanisms of action that need further studies.

A positive correlation was actually found between glucose levels and BPA and BPS liver concentrations, thus suggesting that bisphenol may affect other endocrine systems than the estrogenic one.

As regards genotoxic effects, both BPA and BPS induced an increase in micronuclei (MN) in peripheral blood erythrocytes after 2 weeks of exposure, with respect to controls and BPA high dose induced an increase of micronucleated cells even after 8 weeks. These findings are in agreement with studies which suggested that BPA exposure can lead to aneuploidy through disruption of meiotic process (86, 87) and also genomic structural aberrations like DNA breakage (88). Chromosomal damage, as a result of inefficient or incorrect DNA repair, occurs during the cell division and represents an index of accumulated genotoxic effects (65). A few studies have investigated the mechanism behind the genotoxic action of BPA; Ito and colleagues (89) showed that BPA binds directly to DNA-PKcs, a critical enzyme involved in repair of DNA double-strand breaks, inhibiting its kinase activity. This finding suggests that BPA may affect DNA repair or recombination. A previous study showed that after treatment with BPA, a statistically significant increase in the micronucleus frequency in CHO cells was observed after 24 h exposure to doses of 80 and 120 M (90). Gajowik and co-authors (91) found that, a 2 week exposure with 10 and 20 mg/kg BPA, increased the number of MN in peripheral blood reticulocytes, compared to the control group. Both doses caused nearly twice as many micronuclei as in controls. Moreover, a study performed by Aghajanpour-Mir and colleagues (92) showed clearly observable chromosome aneuploidy or numerical aberration in MCF-7 cell line in amniocytes after exposure with different concentrations of BPA. Both BPA and BPS are also known to disrupt centrosome function and microtubule organization, displaying the broadest spectrum of cancer-promoting effects (93). BPS is indicated as able to elicit a stronger reproductive and DNA damage repair gene response than BPA, being the suppression of msp-50 and msp-152 gene expression following BPS and BPA exposure likely involved in impairing chromosome morphogenesis in diakinesis, besides controlling oocyte maturation and embryonic viability (42). Looking at Cytome data, both bisphenol compounds seem to interfere with cell proliferation. Indeed, a significant reduction in binucleated cell frequency was observed after treatment with both BPA and BPS, at the longest times of exposure. This finding is in agreement with the results of Lee and colleagues (94): mutant chicken DT40 cell lines (deficient in DNA repair pathways) exposure to BPA and BPS resulted in 252 and 9-fold reduction of proliferation in RAD54−/− compared to that of in *wild-type*. The authors also highlighted that cellular proliferation of RAD54/significantly decreased after 48 h exposure to 62.5 µM BPA and 250 µM BPS. The present data could be explained referring to the tendency of bisphenol to interact with cell cycle inhibiting cell division.

There is a scarcity of data available in the literature regarding BPS genotoxicity, since this compound has been introduced only recently as an alternative to BPA. A lower incidence of double strand breaks induced by BPS in comparison with BPA was observed in chicken DT40 cells, but it should be noted that sufficient number of cells could not be identified at the maximum doses of BPS, probably because of considerable decrease in cell proliferation (94). On the other hand, DNA double strand breaks, evaluated by the formation of γ-H2AX foci, a marker correlated with micronuclei formation (95), were induced by BPA but not by BPS in human hepatocellular carcinoma cells. Our data actually indicated BPS as a weaker inducer of MN, in comparison with BPA. In fact, only the highest dose of exposure (20 mg/kg) at the shorter time of exposure (2 weeks) induced a significant increase in the frequency of micronucleated cells, together with an increase of T3. This genotoxic effect could be due to the potential of BPS to induce oxidative stress both *in vivo* (96, 97) and *in vitro* (98, 99). A modified version of the Comet assay demonstrated that BPS induced a genotoxic effect in human prostatic cells and that such effects were strictly associated to oxidative stress mechanisms (52).

Taking into consideration the whole set of data, the present study depicts a scenario where BPA shows its genotoxic and estrogenic potential immediately after 2 week exposure, giving rise to an increase MN frequency after the treatment with the two doses and an increase of VTG levels after the exposure to the high dose. Also the chromosomal alteration persists after 8 weeks, when BPA also interferes with cell cycle inhibiting cell division. The positive correlation found between glucose level and BPA/BPS liver concentrations suggests that BPA and BPS may affect other endocrine systems than the estrogenic one. In particular, the high dose of BPS was found to induce an increase of T3 plasma levels, of chromosomal damage and gene amplification after 2 week exposure, and a cell proliferation impairment after 8 week exposure.

A possible link between BPS-induced genotoxicity and oxidative stress might be mediated by the alteration of metabolic status, as showed by the increase of T3 plasma levels and the subsequent glucose concentration. The increased glucose levels, *via* oxidative mechanisms (100) may be responsible for the onset of chromosomal abnormalities, depicting a scenario where hormonal and metabolic alteration can produce adverse effect at DNA level, which persist longer for BPA. The interference of cell proliferation by BPA and BPS after chronic exposures deserves further attention. 

In conclusion, the present *in vivo* study suggests that BPS may have adverse effects in fish such as *Salmo trutta*, adding to the growing knowledge of the possible risks of using BPS as an alternative to BPA. Results show that BPS might affect thyroid function and glucose balance in the exposed fish. In contrast, BPA showed stronger estrogenic and mutagenic capacity compared to BPS. Taken together, results show that both compounds have adverse effects in exposed fish, although suggesting two different mode of actions. More research concerning the different aspects of BPA and BPS toxicity needs to be performed to obtain a broader as well as more detailed picture of their metabolic/endocrine disruption effects and DNA damaging properties, contributing to evaluate the potential risks for the environment and human health.

## Data Availability Statement

The raw data supporting the conclusions of this article will be made available by the authors, without undue reservation.

## Ethics Statement

The study was performed under the ethical permit (274–2011) issued by the regional committee for animal experiments following national and European ethical guidelines and legislations on animal experimentation under the EU directive 2010/63/EU.

## Author Contributions 

GF: supervision, investigation, writing—original draft, writing—review and editing. JM: investigation, writing—review and editing. MB: investigation, writing—original draft, writing—review and editing. VS: investigation. EJ: conceptualization, investigation, supervision, writing—review and editing. ND: investigation, writing—review and editing. MM: investigation. PG: investigation, writing—original draft, writing—review and editing. JS: conceptualization, funding acquisition, investigation, supervision, writing—review and editing. NA: conceptualization, funding acquisition, investigation, supervision, writing-review and editing. All authors contributed to the article and approved the submitted version.

## Funding

The present project received funding from the Swedish Research Council Formas and the Magnus Bergwall foundation.

## Conflict of Interest

The authors declare that the research was conducted in the absence of any commercial or financial relationships that could be construed as a potential conflict of interest.
